# A Quasiphysics Intelligent Model for a Long Range Fast Tool Servo

**DOI:** 10.1155/2013/641269

**Published:** 2013-09-18

**Authors:** Qiang Liu, Xiaoqin Zhou, Jieqiong Lin, Pengzi Xu, Zhiwei Zhu

**Affiliations:** ^1^College of Mechanical Science and Engineering, Jilin University, Changchun 130022, China; ^2^College of Electromechanical Engineering, Changchun University of Technology, Changchun 130012, China

## Abstract

Accurately modeling the dynamic behaviors of fast tool servo (FTS) is one of the key issues in the ultraprecision positioning of the cutting tool. Herein, a quasiphysics intelligent model (QPIM) integrating a linear physics model (LPM) and a radial basis function (RBF) based neural model (NM) is developed to accurately describe the dynamic behaviors of a voice coil motor (VCM) actuated long range fast tool servo (LFTS). To identify the parameters of the LPM, a novel *Opposition-based Self-adaptive Replacement Differential Evolution (OSaRDE)* algorithm is proposed which has been proved to have a faster convergence mechanism without compromising with the quality of solution and outperform than similar evolution algorithms taken for consideration. The modeling errors of the LPM and the QPIM are investigated by experiments. The modeling error of the LPM presents an obvious trend component which is about ±1.15% of the full span range verifying the efficiency of the proposed OSaRDE algorithm for system identification. As for the QPIM, the trend component in the residual error of LPM can be well suppressed, and the error of the QPIM maintains noise level. All the results verify the efficiency and superiority of the proposed modeling and identification approaches.

## 1. Introduction

In recent years, the fast tool servo (FTS) based single point diamond turning (SPDT) has been gaining increasing applications in extensive areas such as dynamic compensation of machining errors, generation of nonaxisymmetric optical and components, fabrication of microstructured functional surfaces [1–3]. The determination of trajectory tracking strategy for the cutting tool is an ongoing topic for the FTS system to achieve excellent control performance with high accuracy and high robustness, accordingly to achieve fine profile accuracy and high quality of finished surface. 

Up to now, various trajectory tracking strategies have been developed. They could be mainly categorized into model-based control approaches relying on the models of the controlled systems and adaptive control approaches with no dependences on the models. As for the adaptive control approaches, the slide mode controller (SMC) has been mainly implemented for the tool positioning of FTS [4, 5]. The SMC does not need accurate model of controlled system and has relatively high robustness to external disturbances and model uncertainties or variations, but only at the expense of positioning accuracy. The model-based controllers which have been applied to FTS generally comprise of proportional integral derivate (PID) controller [6–8], adaptive feedforward cancellation (AFC) controller [3, 9, 10], repetitive controller (RC) [11, 12], and active disturbance rejection controller (ADRC) [13, 14]. To further enhance the robustness and the stability of FTS system, extended state observer (ESO) based compensation approach serving as one of the key components of the ADRC has been extensively embedded in both model-based controllers and adaptive controllers [10, 11, 13–15]. As for the optimal design process of the PID controller, the feedforward component and phase advance parameters of AFC controller, and the extensively employed ESO compensator, accurate model for describing the physical process of FTS should be critically established. Unfortunately, almost all of the FTSs are approximately equivalent to linear second-order dynamic systems [8–10, 12–14], even for the piezoelectric actuated FTS with inherent hysteresis and creep nonlinearity (HCN) effects [4, 15]. It is evident that there should exist inherent nonlinearities in any practical FTS system, the linear approximations of these servo systems would lead to significant modeling errors and consequently deteriorate control performances of FTS systems. Hence, considerable work should be further done to improve the modeling accuracy.

Generally, the system modeling approaches can be mainly divided into two categories, which are physical approach and phenomenological approach, respectively. On one hand, the description capability of physical model is limited by its assumptions. On the other hand, phenomenological model has its own drawbacks, such as outlier and overfitting. Such problems probably lead to conclusions against physical laws, especially when phenomenological model is used for extrapolation [16–19]. So it is believed that integrating these models has the potential to overcome the drawbacks of both physical and phenomenological models [16–19]. 

Motivated by this, a quasiphysics intelligent model (QPIM) integrating a linear physics model (LPM) and a radial basis function (RBF) based neural model (NM) is proposed to accurately describe the dynamic behaviors of a long range FTS (LFTS) driven by a voice coil motor (VCM), and a novel *Opposition-based Self-adaptive Replacement Differential Evolution (OSaRDE)* algorithm is developed for the parameters identification of the LPM governed by physics laws. The remainder of this paper is organised as follows: the mechanical structure and the modeling procedure of the LFTS is introduced in Section 2; the preliminary of the differential evolution (DE) algorithm and the proposed OSaRDE algorithm is introduced in Section 3; the experiment results and corresponding discussions are carried out in Section 4; the main conclusions of this paper are drawn in Section 5.

## 2. Modeling and Identification Procedure of the LFTS

### 2.1. Mechanical Structure of the LFTS

**Figure 1:**
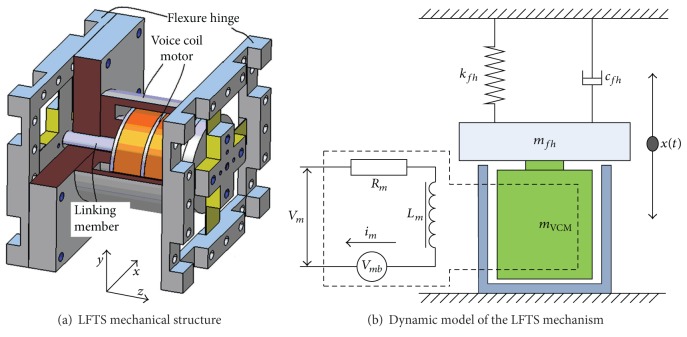
Schematic of the LFTS mechanism.

The authors of this paper have developed a LFTS for ultraprecision diamond turning; the mechanical structure of the LFTS is illustrated in Figure 1(a). The LFTS mainly contains two parts: the VCM actuator and the flexure-based guide mechanism. A BEI Kimco VCM is employed as the driving element. It can provide maximum continuous stall forces of 82.74 N (18.6 lbs) with a stroke of ±5.72 mm. Being benefited from the long stroke of the actuator, the stroke of the LFTS could reach up to 1 mm. Two groups of cross shape flexure hinges are specially designed for the guide of the moving component of the LFTS. The two hinges are connected by a linking member crossing the VCM stator. The mechanical design process and performance evaluations of this LFTS are detailed in [20].

### 2.2. The Quasiphysics Intelligent Model of the LFTS

From an electromagnetic point of view, the VCM can be considered as an inductance component with a resistance [21, 22]. Meanwhile, from the dynamic point of view, the flexure-based mechanism can be equivalent as a damped mass-spring system [8, 23]. With consideration of the mass of the moving part including the flexure hinges and the bobbin of the VCM, the dynamic model of the LFTS mechanism can be obtained, as illustrated in Figure 1(b). The electromagnetic dynamic balance of the VCM in the LFTS, as equivalently shown in Figure 1(b), can be derived based on the Kirchhoff's law, yielding [21, 22]

(1) $$\begin{array}{c} V_{m}=R_{m}i_{m}+L_{m}\frac{di_{m}}{dt}+V_{mb}=R_{m}i_{m}+L_{m}\frac{di_{m}}{dt}+K_{mvs}\frac{dx}{dt}, \end{array}$$

where *V*_*m*_ is the voltage applied to the VCM, *V*_*mb*_ is the back electromotive force (EMF), and *K*_*mvs*_ is the equivalent back EMF constant. *R*_*m*_, *L*_*m*_, and *i*_*m*_ represent the equivalent resistance of voice coils, inductance and applied current of coil, respectively.

With the electrical dynamics in (1) derived, the Flemining's left-hand rule is then employed to derive the electromagnetic forces for actuation, which can be expressed as [21, 22]

(2) $$\begin{array}{c} F=n_{m}B_{m}l_{m}i_{m}=\frac{n_{m}B_{m}l_{m}}{R_{m}}\left(V_{m}-L_{m}\frac{di_{m}}{dt}-K_{mvs}\frac{dx}{dt}\right), \end{array}$$

where *n*_*m*_ is the number of coil loops, *B*_*m*_ is the magnetic flux densities within the air gap between bobbin and magnets, and *l*_*m*_ is the effective coil lengths considering all loops. Generally, the term *L*_*m*_ *di*_*m*_/*dt* can be considered negligible due to the fact that the electrical dynamics of the conducted current is much faster than mechanical behaviors; thus

(3) $$\begin{array}{c} F=n_{m}B_{m}l_{m}i_{m}=\frac{n_{m}B_{m}l_{m}}{R_{m}}\left(V_{m}-K_{mvs}\frac{dx}{dt}\right). \end{array}$$

Based on Newton's second law of motion, the differential equation of dynamic motion for the LFTS mechanism can be given as follows:

(4) $$\begin{array}{c} \left(m_{fh}+m_{\text{VCM}}\right)\frac{d^{2}x}{dt^{2}}+c_{fh}\frac{dx}{dt}+k_{fh}x=F, \end{array}$$

where *m*_*fh*_ and *m*_VCM_ represent the equivalent moving mass of the flexure-based mechanism and the bobbin of the VCM, respectively. *c*_*fh*_ and *k*_*fh*_ represent the equivalent damping coefficient and the equivalent stiffness of the flexure-based mechanism, respectively. 

Submitting (3) into (4) yields

(5) (mfh+mVCM)d2xdt2+(cfh+KmvsnmBmlmRm)dxdt+kfhx  =nmBmlmRmVm.

If this mechanical system is a linear time-invariant (LTI) system, the transfer function of this LFTS system can be obtained as follows by applying the Laplace transform:

(6) $$\begin{array}{c} P\left(s\right)=\frac{X\left(s\right)}{V\left(s\right)}=\frac{K}{s^{2}+2\zeta \omega_{n}s+\omega_{n}^{2}}, \end{array}$$

where *K* = *n*_*m*_ *B*_*m*_ *l*_*m*_/(*m*_*fh*_ + *m*_VCM_)*R*_*m*_, 2*ζω*_*n*_ = (*c*_*fh*_ *R*_*m*_ + *n*_*m*_ *B*_*m*_ *l*_*m*_ *K*_*mvs*_)/(*m*_*fh*_ + *m*_VCM_)*R*_*m*_, *ω*_*n*_^2^ = *k*_*fh*_/(*m*_*fh*_ + *m*_VCM_).

Unfortunately, due to manufacturing tolerances on various crucial dimensions of both bobbin and magnets and possible misalignments of magnetic components, there often exist an uneven magnetic filed within the air gap, as a result, it would lead to a more complex and position-varying magnetic flux density *B*_*m*_ [21, 22]. On the other hand, the temperature in the VCM would increase with the motion ongoing, consequently leads to the variations of the equivalent resistance and inductance of the VCM. Meanwhile, the equivalent damping coefficient and stiffness of the flexure-based mechanism could not be permanent constants due to the inherent properties of the employed material for the mechanical structure. Let x*(*t*) be the real output of the LFTS; it would be difficult for the description of x*(*t*) due to the presence of noise (uncontrollable) factors, measurement error, and inherent nonlinearities of system. Hence, all the factors making the system to be nonlinear and random would significantly deteriorate the accuracy of the LTI model. 

For nonlinear dynamic modelling, neural model has been an extremely popular choice due to its high adaptability to various nonlinear systems [24–27]. In the feedforward neural networks, RBF neural networks are local neural networks with series advantages of the faster training speed, small computing, and simple structure [27]. To enhance the accuracy of the pure physics model as obtained in (6), an RBF neural network (RBFNN) based NM is further employed to model the components with nonlinearities and uncertainties. By introducing this NM, the governing equation of the QPIM for the LFTS mechanism in the time domain can be modified as

(7) $$\begin{array}{c} x^{*}\left(t\right)=L^{-1}\left(X\left(s\right)\right)+NN\left(V_{m},{\dot{V}}_{m}\right), \end{array}$$

where *L* ^−1^(·) represent the inverse Laplace transform operation and *NN*(·) represent the RBF based NM. The command signal and its differentiation are employed as the input of the NM, which are three-layer feedforward networks consisting of an input layer, a hidden layer, and an output layer. Generally, the Gaussian function is employed as the basis function of the hidden layer, given by [27]

(8) $$\begin{array}{c} F_{i}=\exp\left(-\frac{{||x_{i}-c_{i}||}^{2}}{2\sigma_{i}^{2}}\right), \end{array}$$

where **x**_*i*_, **c**_*i*_, and *σ*_*i*_ are the input vector, centre vector, and width of the Gaussian, respectively. ||·|| denotes the Euclidean norm operation. 

The output of the NM is a linear combiner, which is defined by [27]

(9) $$\begin{array}{c} NN\left(V_{m},{\dot{V}}_{m}\right)=\overset{n_{c}}{\underset{i=1}{\sum }}w_{i}\cdot \Phi_{i}\left(V_{m},{\dot{V}}_{m}\right), \end{array}$$

where *w*_*i*_ is the weight between the hidden layer and the output layer; *n*_*c*_ is the number of nodes in the hidden layer.

### 2.3. Parameters Identification Procedure

**Figure 2:**
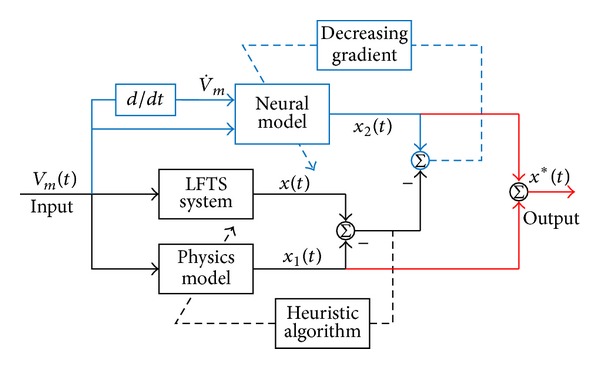
The principle of system identification.

The basic idea in the LFTS system identification is to compare the input dependent response of the system and a corresponding mathematic model by certain performance criterions giving a measure to how well the model response fits the system response. The principle of the identification procedure for the parameter identification of the LFTS system is illustrated in Figure 2, where *x*(*t*) denotes the response of the LFTS system; *x*_1_(*t*) and *x*_2_(*t*) denote the response of the LPM and the NM, respectively. As discussed above, the LPM depends on a set of three parameters, that is, Γ = [*ζ*, *ω*_*n*_, *K*] ∈ *ℜ* ^3^. Hence, the objective of the LPM identification is to find a set of parameters Γ* = [ζ*, *ω*_n_*, K*] ∈ *ℜ* ^3^ that minimize the difference between the response of the real LFTS system and the output of the LPM at each discrete sampling point. Therefore, the identification process lies on minimizing the following criterion:

(10) $$\begin{array}{c} E\left(\Gamma \right)=\overset{N}{\underset{i=1}{\sum }}||x_{1}\left(t_{i}\right)-x\left(t_{i}\right)||, \end{array}$$

where *i* represents the index of the *i*th discrete sampling point. 

The criterion *E*(·) is commonly called a fitness function or objective function reflecting the goodness of solution in the parameter tuning process. The identification problem thus can be treated as a linearly constrained multidimensional optimization problem and formalized as

(11) minimize E(Γ)=∑i=1N||x1(Γ,ti)−x(ti)||, Γ=[ζ,ωn,K]s.t.      Γ∈B, B={Γ,Γmin⁡,j<Γj<Γmax⁡,j,               ∀j=1,2,3},

where **B** is the 3-dimensional feasible search space; Γ_min⁡,*j*_ and Γ_max⁡,*j*_ denote the upper bounds and the lower bounds of the *j*th parameter Γ_*j*_, respectively. 

*E*(Γ) serving as objective function maps decision variables into the objective space. Obviously, the fitness landscape of this identification task may have many local optima and a highly complex topology. Hence, an improved OSaRDE algorithm, which is a simple yet efficient heuristic approach, is employed as the searching tool and will be detailed in the following section.

As for the NM, the similar minimization criterion is defined as

(12) $$\begin{array}{c} N_{E}\left(w\right)=\frac{1}{N}\overset{N}{\underset{i=1}{\sum }}||E\left(\Gamma^{*},t_{i}\right)-x_{2}\left(w,t_{i}\right)||. \end{array}$$

For the training process, the command signal and its differentiation are employed as the input signal, while the residual error of the LPM is employed as the target signal. To obtain the optimal weights of the RBFNN, the developed OSaRDE algorithm could also be implemented as shown in [26]. However, the training of the NM to obtain the optimal weights and structure is executed through the NEURB function in Matlab just for simplicity. Following the scheme provided by Matlab, new neuron is created at each iteration, and the criterion of the new network is checked. If it is low enough, the training is finished; otherwise, the next neuron is added. This procedure is repeated until the criterion goal is met or the maximum number of iteration is reached [27].

## 3. The Opposition-Based Self-Adaptive Replacement Differential Evolution Algorithm

The DE algorithm proposed by Storn and Price (1997) is a heuristic approach for minimizing or maximizing possibly nonlinear and nondifferentiable continuous space functions [28]. Due to some attractive characteristics such as simple differential operator, one-to-one competition scheme, and constructive cooperation between current individuals and memory of the best individuals, DE has been extensively implemented for the identification of both linear and nonlinear systems [29–31]. The obtained results demonstrate that DE is a very promising tool for optimal parameters determination and outperforms other evolutionary technologies [30–32].

### 3.1. The Classical DE Algorithm

The DE algorithm is a population-based algorithm like general evolution algorithms using the similar operators: crossover, mutation, and selection. In DE, a population of potential solution vectors is initialized covering the entire parameter space at the start, which is then evolved to find optimal solutions through the mutation, crossover, and selecting operation procedures [28, 31]. More specifically DE's basic strategy can be formalized as follows.

*(S1)  Initialization.* Assuming that an optimization task consists of *D* parameters, let **B** ∈ *ℜ* ^*D*^ be the search space of the problem under consideration; the initial vector population randomly covering the entire parameter space can be expressed as

(13) $$\begin{array}{c} X_{i}^{0}=\left[x_{i,1}^{0},x_{i,2}^{0},x_{i,3}^{0},\ldots ,x_{i,D}^{0}\right]\in B\;i=1,2,\ldots ,\text{NP}, \end{array}$$

where NP denotes the population size;  **X**_*i*_^0^ denotes a potential solution for the task at start.

*(S2)  Mutation.* For each target vector **X**_*i*_^*k*^ at the *k*th generation, an associated mutant vector ${\hat{X}}_{i}^{k}=({\hat{x}}_{i,1}^{k},{\hat{x}}_{i,2}^{k},{\hat{x}}_{i,3}^{k},\ldots ,{\hat{x}}_{i,D}^{k})$ should be generated via a certain mutation operator. The objective of mutation is to enable search diversity in the parameter space as well as to direct the existing object vectors to better results at a suitable time. Motivated by this, various mutation strategies have been proposed, and certain typical strategies can be summarized as [33, 34]

(14) $$\begin{array}{c} \text{DE}/\text{rand}/1:{\hat{X}}_{i}^{k}=X_{r1}^{k}+F\left(X_{r2}^{k}-X_{r3}^{k}\right), \end{array}$$

(15) $$\begin{array}{c} \text{DE}/\text{best}/1:{\hat{X}}_{i}^{k}=p_{\text{best}}^{k}+F\left(X_{r1}^{k}-X_{r2}^{k}\right), \end{array}$$

(16) DE/cur-to-best/1:X^ik=Xik+F(pbestk−Xik) +F(Xr1k−Xr2k),

where *r*1, *r*2, and *r*3 represent the random and mutually different integers generated within the range [1, NP], and also different from index *I*; *F* is a mutation scale factor within the range [0,2], usually less than 1; **p**_best_^*k*^ denotes the best individual in generation *k*.

*(S3)  Crossover.* After the mutation phase, the crossover operation is applied to each pair of the generated mutant vector ${\hat{X}}_{i}^{k}$ and its corresponding target vector **X**_*i*_^*k*^ to generate a trial vector **Y**_*i*_^*k*^ as follows:

(17) $$\begin{array}{c} Y_{i}^{k}=\left(y_{i,1}^{k},y_{i,2}^{k},y_{i,3}^{k},\ldots ,y_{i,D}^{k}\right),\\ y_{i,j}^{k}=\{\begin{array}{cc} {\hat{x}}_{i,j}^{k}, & \text{if}\;\left(\text{rand}\left(j\right)\leq \text{CR}\;\text{or}\;j=\text{rand}\;n\left(i\right)\right),\\ x_{i,j}^{k}, & \text{if}\;\left(\text{rand}\left(j\right)>\text{CR}\;\text{or}\;j\neq \text{rand}\;n\left(i\right)\right), \end{array} \end{array}$$

where *j* = 1,2,…, *D*; *i* = 1,2,…, NP; rand(*j*) is the *j*th independent random number uniformly distributed in the range of [0,1]. rand *n*(*i*) is a randomly chosen index from the set {1,2,…, *D*}; CR is a user-specified crossover factor within [0,1] that controls the diversity of the population [28, 31].

*(S4)  Selection.* DE employs a greedy selection process that the better one of new offspring and its parent win the competition. If *F*_*C*_(·) denotes the objective function under consideration, the next generation can be determined by

(18) Xik+1={Yik,if  FC(Xik)<FC(Yik),Xik,otherwise.

For the minimum problem, the current best individual **p**_best_^*k*^ at the *k*th generation is defined as [35]

(19) $$\begin{array}{c} p_{\text{best}}^{k}:=\underset{X^{k}}{\arg}\min\left\{F_{C}\left(X_{i}^{k}\right),\;\;\forall i\right\};\; \end{array}$$

**g**_best_^*k*^ which denotes the best individual in the previous entire generations is defined as [35]

(20) $$\begin{array}{c} g_{\text{best}}^{k}=\{\begin{array}{cc} p_{\text{best}}^{k}, & \text{if}\;g_{\text{best}}^{k-1}>p_{\text{best}}^{k},\\ g_{\text{best}}^{k-1}, & \text{otherwise}. \end{array} \end{array}$$

### 3.2. The Improved OSaRDE Algorithm

However, the optimum performances of different problems are not only highly dependent on the control parameters and learning strategies involved in DE, but also dependent on the characteristics of the region of the search landscape being explored [36, 37]. For a given task, we should spend a huge amount of time to try through various strategies and fine tune the corresponding parameters to achieve the best optimum performance. Motivated by this dilemma, large number of researchers have devoted themselves to develope adaptive DE algorithm to solve general problems more efficiently. Up to now, a classification, into three macrogroups of the main adaptive strategies employed in the DE scheme can be proposed here. (1) Self-adapting parameter setting of *F* and CR, including the pseudorandom based updating approach [34, 36, 38] and the local optimum search based updating approach [39, 40]. (2) Adaptive selection of mutation strategies according to their capacities of generating promising solutions [36, 37, 41]. (3) Dynamic population sizing strategy based on self-adaptation [42] or progressive reduction [43, 44]. 

The classical DE algorithm typically converges at a rapid pace in the initial stages of the search procedure and then gradually slows down or keeps unchanged as it approaches global optimum. In case there is no improvement in the fitness in successive generations, it would be an indication that the individuals are clustered together in a local region and the operations are very insignificant to allow any improvement of the evolution process [45]. As is evident from this phenomenon, the information of the evolutionary process contained in these individuals would overlap and become extensively redundant. However, we do not suggest that we should discard the redundant individuals by the dynamic population sizing strategy. If the redundant individuals were simply discarded, the information contained in these individuals would be totally lost. Moreover, the population sizing strategy also has no assistance to the performance of escaping from local attractors. 

In order to avoid the redundancy and enhance the population diversity, Ali and Pant (2011) take advantage of Cauchy mutation to perturb the clustered individuals and force them to move to someother location, thereby providing them an opportunity to improve their performance [45]. Being totally different from Cauchy mutation based and other adaptive strategies based DE algorithm mentioned above, a novel *Opposition-based Self-adaptive Replacement Differential Evolution (OSaRDE)* algorithm is proposed in this research to enhance the performance of DE. In the OSaRDE scheme, the redundant inferior individuals are self-adaptively selected according to a criterion defined by Euclidean distance after a specified number of generations, and then each of them is replaced by its opposite based on the opposition-based learning (OBL) strategy. After a period of regular evolutions, the process will be repeated. More specifically OSaRDE's basic strategy considering the constraint task will be formalized as follows.

#### 3.2.1. The OSaRDE's Basic Strategy

For the replacement operation in the *k*_*n*_^*R*^ generation, the current worst individual **p**_worst_^*k*_*n*_^*R*^^ can be defined as

(21) $$\begin{array}{c} p_{\text{worst}}^{k_{n}^{R}}:=\underset{X^{k_{n}^{R}}}{\arg}\max\left\{F_{C}\left(X_{i}^{k_{n}^{R}}\right),\;\;\forall i\right\}. \end{array}$$

The redundant inferior individuals are defined as

(22) UmknR=XiknR if(||XiknR−pworstknR||≤Θ·Dis),Dis=||pbestknR−pworstknR||,i=1,2,…,NP,  m=1,2,…,M,    n=1,2,…,N,

where *M* denotes the number of inferior individuals, which is self-determined according to (22); Θ representing the replacement ratio is a user-defined constant; ||·|| denotes the operator for Euclidean distance; *N* denotes the total number of the replacement operations during the evolution process. Let *T*, *T*_*C*_, and *T*_*P*_ represent the number of total generations, the specified number of generations from which the replacement operation begins, and the evolution period during two adjacent replacement operations. Considering that the replacement operation should not be executed at the last generation, *N* can be obtained as

(23) $$\begin{array}{c} N=\text{ceil}\left(\frac{T-T_{C}}{T_{P}}\right)-1. \end{array}$$

The opposite of each selected inferior individual can be defined as [26, 46]

(24) $$\begin{array}{c} OU_{m}^{k_{n}^{R}}=MIN^{P}+MAX^{P}-U_{m}^{k_{n}^{R}}, \end{array}$$

where **M** **I** **N** ^**P**^ = [min_1_^*p*^, min_2_^*p*^,…, min_*D*_^*p*^] and **M** **A** **X** ^**P**^ = [max_1_^*p*^, max_2_^*p*^,…, max_*D*_^*p*^] represent the predefined boundaries of the optimum task.

By applying the replacement operation, the population in the *k*th generation can be obtained as

(25) $$\begin{array}{c} X_{i}=\{\begin{array}{cc} OU_{m}^{k_{n}^{R}} & \text{if}\;\left(||X_{i}^{k_{n}^{R}}-p_{\text{worst}}^{k_{n}^{R}}||\leq \Theta \cdot \text{Dis}\right).\\ X_{i} & \text{otherwise}. \end{array} \end{array}$$

By applying the replacement approach to the self-adaptively selected inferior individuals, the evolutionary process dynamically calculates the corresponding opposite according to the current location of each selected individual, thereby forcing them to jump to new solution candidates. The evolution process would possess much higher diversity and accordingly escape from the problem of premature convergence. Then the evolution process could have more opportunities to find better individuals during a period of regular evolutions after a replacement operation.

#### 3.2.2. The Constraint Handling Technique

Generally, most of the optimum problems in engineering application are constrained. Unfortunately, the constraint of the searching space is not considered in the conventional DE scheme. A simple and popular repair operation of the individuals beyond the boundary works as follows [47]: if the *j*th element ${\hat{x}}_{i,j}^{k}$ of the mutant vector ${\hat{X}}_{i}^{k}$ is out of the search region [min_*j*_^*p*^, max_*j*_^*p*^], then ${\hat{x}}_{i,j}^{k}$ is reset as

(26) $$\begin{array}{c} {\hat{x}}_{i,j}^{k}=\{\begin{array}{cc} \min\left\{\text{ma}{\text{x}}_{j}^{p},2\text{mi}{\text{n}}_{j}^{p}-{\hat{x}}_{i,j}^{k}\right\} & \text{if}\;\left({\hat{x}}_{i,j}^{k}<\text{mi}{\text{n}}_{j}^{p}\right),\\ \max\left\{\text{mi}{\text{n}}_{j}^{p},2\text{ma}{\text{x}}_{j}^{p}-{\hat{x}}_{i,j}^{k}\right\} & \text{if}\;\left({\hat{x}}_{i,j}^{k}>\text{ma}{\text{x}}_{j}^{p}\right). \end{array} \end{array}$$

For sake of clarity, the framework of the proposed OSaRDE is emphasized in Algorithm 1.

### 3.3. A Brief Testing on Benchmark Functions

A comprehensive set of benchmark functions, including eight typical global optimization problems, has been used only for performance verification and comparison of the proposed OSaRDE. The definition of the selected benchmark functions and their global optimum(s) are listed in the appendix [45, 48].

The eight algorithms described in the appendix have been tested and compared with standard DE and Cauchy mutation based DE (MDE) proposed by Ali and Pant (2011) [45]. Experiments related to DE and MDE in [45] have been employed as a reference. In order to have a fair comparison, the control parameters of OSaRDE are set as constant, where *F* = 0.7 and CR = 0.3, which are the same as employed in [45]. The number of function evaluations (NFEs) of OSaRDE are kept as 1 × 10^5^, which is half of that employed in [45]. The DE/cur-to-best/1 is chosen as the mutation strategy. In order to have statistically significant results, each test has been run 30 times. The average absolute error and standard deviation are recorded for comparisons. The corresponding results are given in Table 1, and the best results are highlighted in bold face. As evidence from the results in Table 1, it can be clearly observed that both DE and MDE algorithms give the same results for lower dimension problem, while almost all of the absolute errors obtained by OSaRDE are significantly smaller than that obtained by DE and MDE except for *f*_SWF_(**X**). As for *f*_SWF_(**X**), the average absolute error obtained by OSaRDE is larger than that obtained by MDE, while it is quite smaller than those obtained by DE. The results demonstrate that the proposed OSaRDE has a faster convergence mechanism without compromising with the quality of solution, significantly outperforming DE and MDE for both low and high dimension optimum tasks. It could be a fine choice for the OSaRDE to be applied to the engineering practice.

## 4. Experiment Results and Discussion

**Figure 3:**
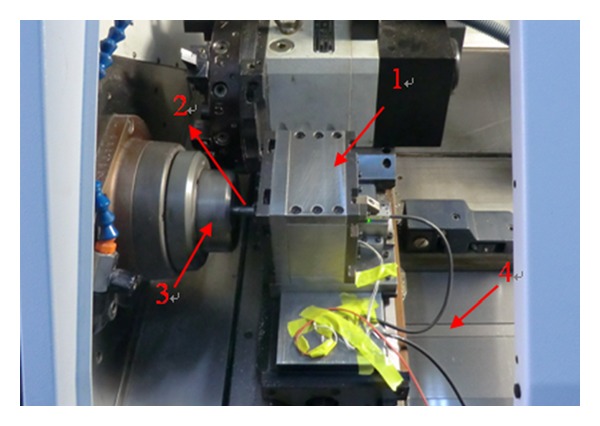
The testing and working condition of the LFTS. ((1) The developed LFTS; (2) the workpiece; (3) the spindle; (4) the lathe bed.)

Figure 3 illustrates the testing condition of the experiment part, where the LFTS is installed on a precision machine tool. The command signal to be applied to the LFTS is generated by a computer and converted through a data-acquisition card from ADLINK. The displacement of the LFTS is measured by a Renishaw linear encoder with a resolution of 10 nm. The measured displacement which is a digital signal is then directly gathered and stored in the computer through the data-acquisition card with a sample time of 0.45 ms for further analysis.

### 4.1. Identification Results of the LPM

**Figure 4:**
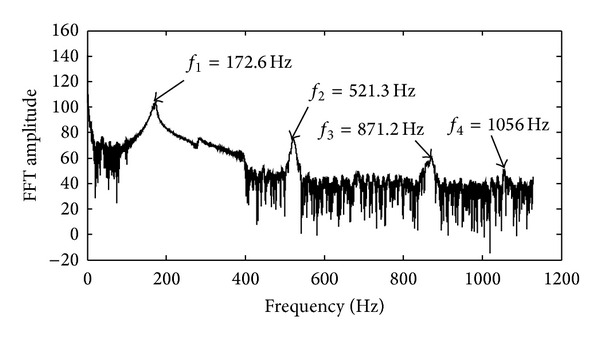
Fourier transform of the response with sweep excitation.

To catch some prior knowledge of the LFTS system and accordingly help achieve more accurate parameters of the system, the sweep check is carried out to estimate the damped natural frequency. Thus, a sweep harmonic signal with constant amplitude and varying frequency ranged from 0 Hz to 400 Hz is applied to the VCM actuator. Fourier transform is then applied to the corresponding response, and the result is illustrated in Figure 4. As shown in Figure 4, the first damped natural frequency is about 172.6 Hz. Generally, the damping factor is about 0.05 and less than 0.1. With the roughly estimated first damped natural frequency and damping factor, the proportionality factor *K* can be investigated as 5 × 10^6^. Thus, the feasible search space of the physical model can be defined as

(27) Γmin⁡=[0,2(1−α)π×172.6,(1−β)×5×105],Γmax⁡=[0.1,2(1+α)π×172.6,(1+β)×5×105],

where *α* = 0.1 and *β* = 0.5 are the scale factors of the first damped natural frequency and damping factor, respectively.

**Figure 5:**
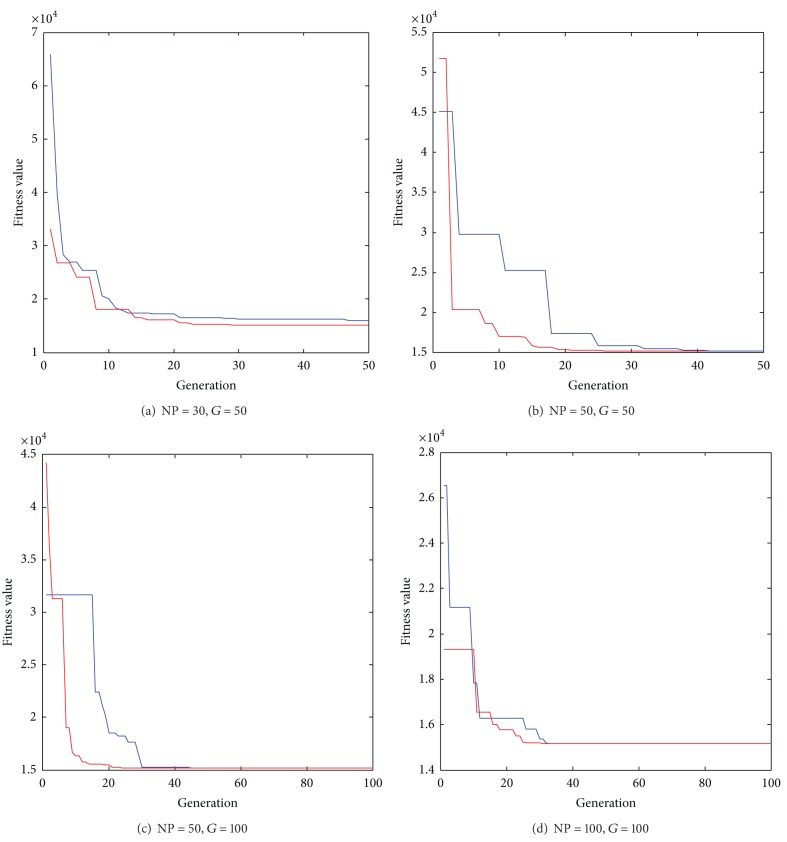
The convergence of the optimum process (the red line and the blue line represent the process of OSaRDE and DE, resp.).

To identify the parameters of the LPM, the step command with 1 V voltage and the corresponding response of the LFTS are employed for the parameter identification due to the reason that the step response contains abundant information of the system dynamics, such as the natural frequency and the damping behavior. To guarantee that the identified results have reached up to the maximum description capacity of the LPM, the optimum procedure is carried out by applying OSaRDE with different population sizes and generations, and the obtained best fitness values are further compared with them obtained by standard DE. The best fitness values are summarized in Table 2, and the convergence processes are illustrated in Figure 5. As given in Table 2, the proposed OSaRDE outperforms DE for low NFEs and gradually tends to be the same for large NFEs from the perspective of best fitness values. It also should be noticed that the best fitness values obtained by OSaRDE with different NFEs remain the same. It means that the OSaRDE is of high robustness for optimum tasks and could afford a much faster convergence rate than DE, thereby effectively reducing the computational time. 

The transfer function of the LFTS can be obtained by introducing the optimum parameters corresponding to the best fitness value, that is

(28) $$\begin{array}{c} G\left(s\right)=\frac{6.19\;\times \;10^{6}}{s^{2}+50.97s+1.196\times 10^{6}}. \end{array}$$

### 4.2. Comprehensive Results of the QPIM

**Figure 6:**
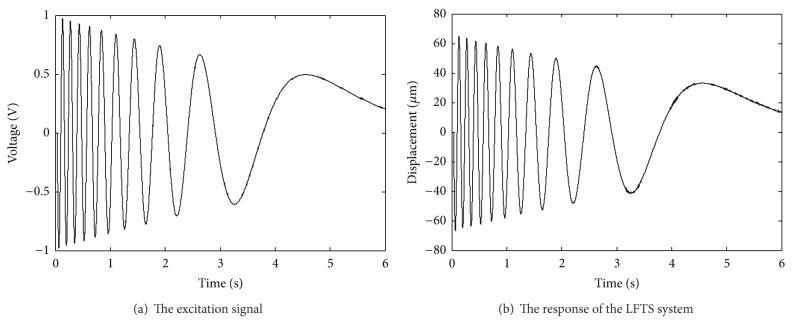
The excitation signal and the corresponding response of the LFTS.

**Figure 7:**
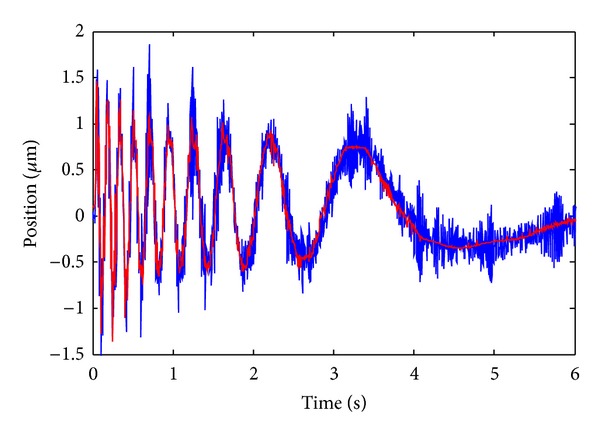
Modeling and prediction results of the NM (the blue line denotes the modeling error of the LPM and the red line denotes the output of the NM).

**Figure 8:**
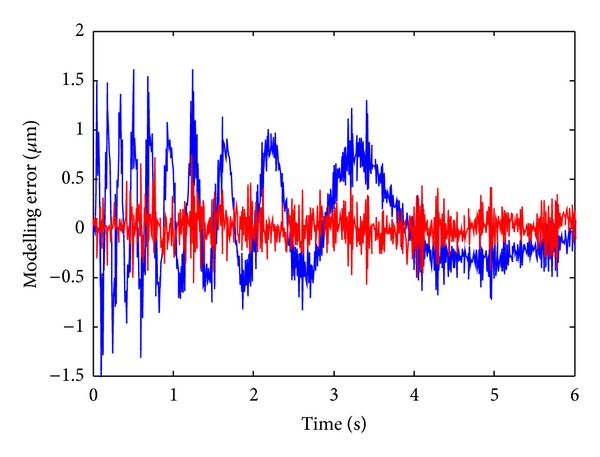
Modeling errors of the LPM and the QPIM (the blue line and the red line denote the modeling error of the LPM and the QPIM, resp.).

In this experiment, an input excitation scheme with varying frequency and amplitude as shown in Figure 6(a) is applied to the VCM, and the corresponding output of the LFTS is illustrated in Figure 6(b). The 13600 sampling data are divided into two parts in which the former 6000 groups of data are used for training the NM and the latter 7600 groups of data are used for prediction. The modeling and prediction performance of the trained RBF NM is illustrated in Figure 7. As is evident from Figure 7, the NM could well approximate to the residual error of the LPM and present a good prediction capacity. To give a comprehensive comparison of the performances between the LPM and the proposed QPIM with neural model calibration, the modeling errors of the two models are illustrated in Figure 8. It can be seen that the maximum modeling error of the LPM is about ±1.15% of the full span range; meanwhile, obvious trend component attributing to the unmodeled parts of the physical process which could not be described by the LPM can be observed. The relatively small error verifies the efficiency of the proposed OSaRDE algorithm for system identification. As for the QPIM, the trend component in the residual modeling error of the LPM is well suppressed and the corresponding error maintains noise level. The result demonstrates that the QPIM is able to coincide more effectively with the response of the LFTS system; namely, the proposed modeling and identification method is of excellent performance.

## 5. Conclusion

A quasiphysics intelligent model (QPIM) integrating a linear physics model (LPM) and a radial basis function neural network (RBFNN) based neural model (NM) is developed in this paper to accurately describe the dynamic behaviors of a voice coil motor (VCM) actuated long range fast tool servo (LFTS). A linear second-order differential equation of the LFTS system is established according to physics laws, and a novel Opposition-based Self-adaptive Replacement Differential Evolution (OSaRDE) algorithm is further developed for the parameter identification of the LPM. The NM is developed for the calibration of the unmodeled parts of the physics process attributing to the nonlinearity effect of the system. Both numerical and experiment examinations are carried out to respectively assess the performance of the OSaRDE algorithm and the QPIM. Certain conclusions can be drawn as follows.

1. A comprehensive set of benchmark functions, including eight typical global optimization problems, has been used for performance verification and comparison of the proposed OSaRDE. In comparison with both standard DE and a modified DE (MDE), it is observed that the OSaRDE performs remarkably better for both low and high dimension optimum tasks. The observation demonstrates that the proposed OSaRDE has a faster convergence mechanism without compromising with the quality of solution and outperforms DE and MDE taken for consideration. We may suggest that it should be a fine choice for the OSaRDE to be implemented for engineering practice.
2. As is evident from the identification results of the LPM and the QPIM, the modeling error of the LPM presents an obvious trend component which is about ±1.15% of the full span range. The relatively small error verifies the efficiency of the proposed OSaRDE algorithm for system identification. The residual modeling error is further reduced by NM calibration; the trend component in the residual error of the LPM is well suppressed, and the error of the QPIM maintains noise level. The result demonstrates that the QPIM is able to coincide more effectively with the response of the LFTS system, namely, the proposed modeling and identification method is of excellent performance.

## Figures and Tables

**Algorithm 1:**
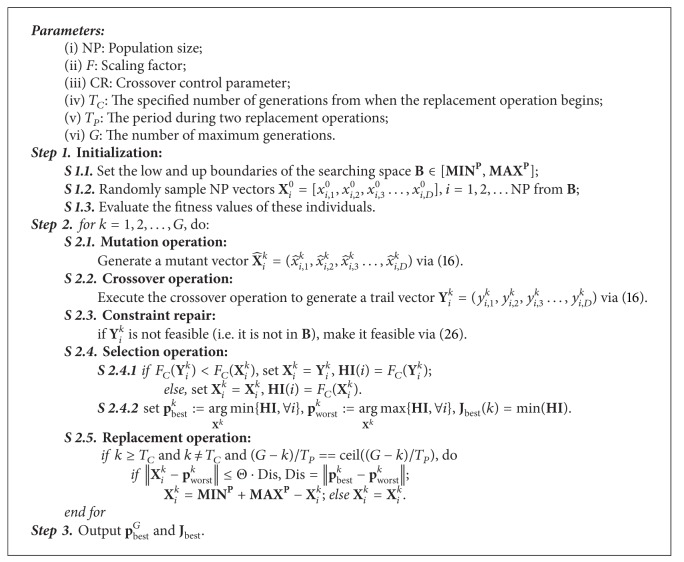
The framework of the OSaRDE.

**Table 1:** Comparisons of DE, MDE, and OSaRDE in terms of average absolute error and standard deviation for 30 runs.

Fun.	*D *	Absolute error			Standard deviation		
		DE	MDE	OSaRDE	DE	MDE	OSaRDE
*f*_*EP*⁡_(**X**)	2	1.3424*e* − 06	1.3424*e* − 06	0.0000**e** − 00	6.9820*e* − 07	1.2595*e* − 06	0.0000*e* − 00
*f*_SP_(**X**)	30	8.2416*e* − 05	9.2528*e* − 07	6.0296**e** − 18	9.7617*e* − 06	1.9492*e* − 07	2.9392*e* − 17
*f*_ACK_(**X**)	30	2.5799*e* − 03	5.5200*e* − 04	2.5364**e** − 10	1.7069*e* − 4	8.7203*e* − 05	9.3680*e* − 10
*f*_GW_(**X**)	30	4.8967*e* − 04	1.8229*e* − 07	3.2746**e** − 09	9.3176*e* − 05	6.7985*e* − 08	1.1802*e* − 08
*f*_LM2_(**X**)	30	9.3176*e* − 05	1.5778*e* − 07	4.8193**e** − 13	1.5190*e* − 06	204184*e* − 08	1.8087*e* − 12
*f*_SWF_(**X**)	30	5.9698*e* + 02	6.8199**e** − 04	2.9260*e* + 02	5.1068*e* + 02	6.8199*e* − 04	1.0148*e* + 01
*f*_RB_(**X**)	30	3.0242*e* + 01	2.4416*e* + 01	2.0256**e** + 01	9.7011*e* − 01	1.5638*e* − 01	8.1206*e* + 00
*f*_RG_(**X**)	30	1.3773*e* + 02	8.6468*e* + 01	8.2609**e** + 01	7.4039*e* − 00	1.2761*e* + 01	7.2384*e* + 00

**Table 2:** Fitness values with different population sizes and generations.

	*G* = 50		*G* = 100	
	*P* = 30	*P* = 50	*P* = 50	*P* = 100
OSaRDE	1.517*e* + 04	1.517*e* + 04	1.517*e* + 04	1.517*e* + 04
DE	1.592*e* + 04	1.519*e* + 04	1.517*e* + 04	1.517*e* + 04

## Appendix

### Benchmark Problems

(1)*  Eason Function (EP):*

(A.1) $$\begin{array}{c} f_{\mathrm{EP}}\left(X\right)=-\cos\left(x_{1}\right)\cos\left(x_{2}\right)\exp\left(-{\left(x_{1}-\pi \right)}^{2}-{\left(x_{2}-\pi \right)}^{2}\right), \end{array}$$

with −10 ≤ *x*_*i*_ ≤ 10 and min⁡*f*_*EP*⁡_(*π*, *π*) = −1.

It is a multimodal, nonseparable function having several optima.

(2)*  Sphere Function (SP):*

(A.2) $$\begin{array}{c} f_{\text{SP}}\left(X\right)=\overset{n}{\underset{i=1}{\sum }}x_{i}^{2}, \end{array}$$

with −100 ≤ *x*_*i*_ ≤ 100 and min⁡*f*_SP_(0,…, 0) = 0.

It is a simple, continuous unimodal, separable, and highly convex function. It serves as test case for validating the convergence speed of an algorithm.

(3)*  Ackley's Function (ACK):*

(A.3) fACK(X)=−20exp⁡(−0.2∑i=1nxi2n) −exp⁡(1n∑i=1ncos⁡(2πxi))+20+e,

with −30 ≤ *x*_*i*_ ≤ 30 and min⁡*f*_ACK_(0,…, 0) = 0.

It has numerous local minima, but its complexity is moderated. Any search strategies that analyzes a wider region will be able to cross the valley among the optima and achieve the global optimum.

(4)*  Griewank Function (GW):*

(A.4) $$\begin{array}{c} f_{\text{GW}}\left(X\right)=\frac{1}{4000}\overset{n}{\underset{i=1}{\sum }}x_{i}^{2}-\overset{n}{\underset{i=1}{\prod }}\cos\left(\frac{x_{i}}{\sqrt{i}}\right)+1, \end{array}$$

with −600 ≤ *x*_*i*_ ≤ 600 and min⁡*f*_GW_(0,…, 0) = 0.

It is a highly multimodal, nonseparable function. It has many regularly distributed local minima. It tests both convergence speed and the stability to escape from a shallow local minimum.

(5)*  Levy and Montalvo 2 Problem (LM2):*

(A.5) fLM2(X)=sin2(3πx1)+∑i=1n−1(xi−1)2(1+sin2(3πxi+1)) +(xn−1)(1+sin2(2πxn)),

with −50 ≤ *x*_*i*_ ≤ 50 and min⁡*f*_GW_(1,…, 1) = 0.

It is a multimodal, nonseparable function having several local optima.

(6)*  Schwefel's Problem (SWF):*

(A.6) $$\begin{array}{c} f_{\text{SWF}}\left(X\right)=418.9829n-\overset{n}{\underset{i=1}{\sum }}x_{i}\sin\left(\sqrt{\left|x_{i}\right|}\right), \end{array}$$

with −500 ≤ *x*_*i*_ ≤ 500 and min⁡*f*_GW_(420.97,…, 420.97) = 0.

It is a multimodal function with very deep sinusoidal interactions. It is generally considered to be difficult for optimization algorithms.

(7)*  Rosenbrock Problem (RB):*

(A.7) $$\begin{array}{c} f_{\text{RB}}\left(X\right)=\overset{n-1}{\underset{i=1}{\sum }}\left[100{\left(x_{i+1}-x_{i}^{2}\right)}^{2}+{\left(1-x_{i}\right)}^{2}\right], \end{array}$$

with −30 ≤ *x*_*i*_ ≤ 30 and min⁡*f*_RB_(1,…, 1) = 0.

As the number of dimensions increase, it ceases to be unimodal. The optimum of RB function lies inside a long, narrow, parabolic-shaped float valley. It helps in testing the ability of an algorithm to prevent premature convergence.

(8)*  Rastrigin's Function (RG):*

(A.8) $$\begin{array}{c} f_{\text{RG}}\left(X\right)=10n+\overset{n}{\underset{i=1}{\sum }}\left(x_{i}^{2}-10\cos\left(2\pi x_{i}\right)\right), \end{array}$$

with −5.12 ≤ *x*_*i*_ ≤ 5.12 and min⁡*f*_RG_(0,…, 0) = 0.

It is a multimodal, nonseparable function having large numbers of local optima.
